# Advances in the Detection of Pancreatic Cancer Through Liquid Biopsy

**DOI:** 10.3389/fonc.2021.801173

**Published:** 2021-12-21

**Authors:** Tian-Bao Yan, Jia-Qi Huang, Shi-Yun Huang, Bhavesh K. Ahir, Long-Man Li, Zeng-Nan Mo, Jian-Hong Zhong

**Affiliations:** ^1^ Department of Hepatobiliary Surgery, Guangxi Medical University Cancer Hospital, Nanning, China; ^2^ Center for Genomics and Personalized Medicine, Guangxi Key Laboratory for Genomics and Personalized Medicine, Guangxi Collaborative Innovation Center for Genomics and Personalized Medicine, Guangxi Medical University, Nanning, China; ^3^ Section of Hematology and Oncology, Department of Medicine, University of Illinois at Chicago, Chicago, IL, United States

**Keywords:** diagnose, liquid biopsy, pancreatic cancer, exosomes, cfDNA (circulating free DNA)

## Abstract

Pancreatic cancer refers to the development of malignant tumors in the pancreas: it is associated with high mortality rates and mostly goes undetected in its early stages for lack of symptoms. Currently, surgical treatment is the only effective way to improve the survival of pancreatic cancer patients. Therefore, it is crucial to diagnose the disease as early as possible in order to improve the survival rate of patients with pancreatic cancer. Liquid biopsy is a unique *in vitro* diagnostic technique offering the advantage of earlier detection of tumors. Although liquid biopsies have shown promise for screening for certain cancers, whether they are effective for early diagnosis of pancreatic cancer is unclear. Therefore, we reviewed relevant literature indexed in PubMed and collated updates and information on advances in the field of liquid biopsy with respect to the early diagnosis of pancreatic cancer.

## Introduction

Pancreatic cancer (PC) refers to malignant tumors of the digestive system originated mainly from pancreatic ductal epithelium and acinar cells. PC is associated with low 5-year survival rates (9%) and worse prognosis (1). Over 90% of PC patients develop pancreatic ductal adenocarcinoma (PDAC) or its variants (2). Data from the International Agency for Research on Cancer indicates that PC is the 12th most common type of cancer reported worldwide and the 7th most common cause of cancer-related deaths (3). The risk factors for PC include cigarette smoking (4), alcohol consumption (5), *Helicobacter pylori* infection (6), previous history of diabetes mellitus (7), and family history of PC (8). Given the range of risk factors associated with this disease, primary prevention is difficult.

Clinical manifestations of PC include dyspepsia, weight loss, nausea, jaundice, vomiting, floating stool, pain, and sometimes, pancreatitis (2). However, most PC patients show no obvious symptoms until the disease reaches an advanced stage: this makes early detection difficult. Although computerized tomography (CT) and magnetic resonance imaging (MRI) are widely available tools that can provide qualitative information for diagnosing PC, there was no significant difference between CT and MRI in the detection of pancreatic cancer (9). Due to its poor sensitivity for detecting small pancreatic lesions, CT must be performed in association with invasive, relatively expensive procedures that are often impractical for screening (10). Moreover, both CT and MRI have limitation for their certain radioactivity (11). Attempts to detect PC early using endoscopic ultrasonography or other imaging techniques have also yielded unsatisfactory results (12).

According to the imaging evaluation, PC can be divided into resectable, borderline resectable, locally advanced, and those with distant metastasis. Systemic therapy improves survival. However, surgical resection is considered the only treatment to cure PC which can help significantly increase the survival of patients with PC. In fact, pancreatectomy can prolong survival of patients with early-stage PC by at least 2 years (13, 14). Although adjuvant chemotherapy after surgery can improve median overall survival compared with no chemotherapy (15–19), only 15-20% of PC patients are candidates for pancreatectomy due to late presentation of the disease (20). Therefore, the early detection of PC is crucial.

Carbohydrate antigen 19-9 (CA19-9) is the most commonly used biomarker for the early detection and monitoring of PC, but it is not specific to that disease (21–23). A better alternative may be to test for more reliable cancer markers by liquid biopsy, a relatively new branch of *in vitro* diagnosis based on analysis of biomarkers in body fluids such as blood, urine, and cerebrospinal fluid (24). This technique was ranked one of the top 10 breakthrough technologies in 2015 by MIT *Technology Review*. Common approaches for cancer screening in the liquid biopsy field have focused on exosomes, circulating tumor DNA (ctDNA), circulating microRNA (miRNA), and circulating tumor cells (CTCs) (25, 26). In patients with PC, the main application of liquid biopsy is to use information collected from blood tests to monitor the developmental process of the tumor. This technique can also be used to collect data on drug resistance and other information that may help guide personalized precision therapies.

Liquid biopsy has many advantages over existing tumor detection methods: it is non-invasive and therefore can be conducted frequently, which allows dynamic monitoring and can overcome the problem of tumor heterogeneity (27–29). The application of liquid biopsy to cancer screening has been mentioned in many official guidelines: for example, the National Comprehensive Cancer Network (NCCN) Guidelines about Pancreatic Adenocarcinoma (Version 1; 2021) has reported promising results when screening for PC based on liquid biopsy biomarkers such as cell-free DNA (cfDNA) and circulating miRNA (2). However, related research content in Guidelines is less described, and clinicians require a better understanding of the advantages of using liquid biopsy to effectively diagnose and treat patients with PC. Therefore, in this review, we provide an update on the current advances in the early detection of PC through liquid biopsy by taking into account the decades of research on PC detection methods and associated treatments.

## Search Methods and Data Extraction

This literature review focused on early diagnosis of PC by liquid biopsy. PubMed database was systematically searched from January 1, 2011 to July 5, 2021 with the following keywords: *pancreatic cancer, screening, liquid biopsy, exosomes, ctDNA, miRNA*, and *circulating tumor cells*. Studies reporting the sensitivity, specificity, AUC value, or p value of liquid biopsy (including exosomes, ctDNA, miRNA, and circulating tumor cells) for early diagnosis of PC would be included into analysis. For studies with overlapping patient samples by same centers and overlapped enrollment period, only the study with the largest sample was included. Three authors (T.-B.Y, J.-Q.H and S.-Y.H) independently assessed the eligibility of studies. They independently extracted the data from included studies. If there were any disagreements during the study evaluation or data extraction process, they were resolved by discussion. The following data were extracted: study information, detection method, comparative cohort, treatment duration, and main findings of liquid biopsy.

## Role of Exosomes in PC

Exosomes are small (30-140 nm), membrane-bound particles that are released into the extracellular environment when large multivesicular bodies fuse with the plasma membrane (26). Exosomes contain specific proteins, lipids, and nucleic acids that can be transmitted as signaling molecules to alter the function of other cells (30). Tumor cells are reported to secrete larger proportions of exosomes than normal cells (31). Therefore, exosomes can be considered potential biological indicators of different types of cancers and can be used for the early detection of PC by testing and analyzing the levels of exosomal proteins, nucleic acids, or both that have been secreted into body fluids.

There is growing evidence that exosomes can act as a potential biomarker of PC. Exosomal proteins, lipids, and nucleic acids may contain genetic information that can indicate early changes of PC. Identifying special molecular species differentially expressed between PC patients and healthy individuals can lead to the definition of biomarkers for the presence and characteristics of PC. We have summarized the literature on the clinical application of exosomal biomarkers for the early detection of PC into three categories.

In the first category, researchers have considered exosomes as a new marker or screening index for PC. Certain exosomal markers have demonstrated higher sensitivity, specificity, and area under the receiver operating characteristic curve (AUC) than the gold standard, which indicated that they could play an important role in the early clinical diagnosis of PC. Other studies have compared the clinical advantages of exosomal markers against CA19-9. For example, Melo et al. (32) used mass spectrometry analysis to show that the proportion of glypican-1 (GPC1), a membrane-anchored protein, was significantly higher in serum samples of PC patients than healthy controls. This study analyzed serum samples to differentiate between 100 healthy individuals and 190 patients, including some who had benign PC and some with early- or late-stage PC. Those investigators were able to detect GPC1^+^ circulating exosomes with sensitivity and specificity of 100%. Lux et al. (33) demonstrated that the expression of the proto-oncogene mesenchymal epithelial transition factor (c-Met) was significantly higher in PDAC patients than in PC patients with benign tumors. Those researchers went on to show that a combination of c-Met and CA 19-9 can increase the specificity of early PC diagnosis to 89.5% without losing sensitivity.

In the second category, researchers have developed “chips” to detect tumor-derived exosomes in blood samples in order to diagnose PC. Based on the principle that signal amplification can be achieved by combining the advantages of quantum dots and bionic periodic nanostructures of photonic crystals, Zhang et al. (34) developed a method to analyze circulating tumor exosomes in serum. Nanosized molecular beacons with high luminescence efficiency were used to detect GPC1, whose signal was then amplified *via* photonic crystals. The method significantly improved the sensitivity and specificity of tumor exosome detection and helped effectively distinguish between serum samples from PC patients or healthy individuals. Lewis et al. (35) used alternating current electrokinetic microarray chips to measure levels of GPC1 and CD63 in whole blood, plasma, or serum samples. Those investigators were able to distinguish between 20 PDAC patients and 11 healthy individuals with sensitivity of 99% and specificity of 82%.

In the third category, researchers have demonstrated that the risk of developing PC can be assessed by detecting DNA mutations, such as in the KRAS and TP53 genes, in circulating exosomes. Yang et al. [jiashang] showed that, of the 48 PDAC patients analyzed, 39.6% had KRAS^G12D^ mutations and 4.2% had TP53 ^R273H^ mutations, while only 2.6% of the 114 healthy controls analyzed presented with KRAS^G12D^ mutations and none had TP53 ^R273H^ mutations. However, Allenson et al. (36) analyzed a validation cohort of 82 healthy controls and 39 PDAC patients and found that 43.6% of early PDAC patients and 20% of healthy controls had mutations in KRAS DNA in exosomes, suggesting that KRAS mutations may not be reliable for early assessment of PC. Therefore further study is needed.

GPC1, in contrast, has shown high sensitivity and specificity for detecting PC (32). Biopsying for GPC1^+^ circulating exosomes may perform better than assaying the commonly used biomarker CA19-9. Early detection of PC using GPC1^+^ circulating exosomes has several advantages, including low cost, requirement for minimal blood, and compatibility with long sample storage. In fact, exosomes have been isolated and analyzed from blood samples that had been in the freezer for 30 years (32). Nevertheless, there is no clear consensus on the clinical application of GPC1 for the detection of PC. On one hand, Lai et al. (28) found no significant difference in GPC1 expression in exosomes from PDAC patients or healthy controls (37), and Xu et al. (38) were unable to reliably measure differential expression of exosome miRNAs in PDAC patients using anti-GPC1 antibodies. On the other hand, a case study associated high levels of circulating GPC1with cystic lesions of the pancreas in a patient who showed malignant progression one year later (39). Thus, the possibility remains that liquid biopsy of GPC1 may be useful for detecting certain events in early-stage PC.

Analysis of tumor-derived exosomes may predict prognosis of patients. For example, overexpression of exmiR-21 appears to correlate with worse prognosis and can be used to distinguish PC patients from tumor-free patients: in one study, the median survival time was significantly longer in the low expression group (846 days) than in the high expression group (344 days) (40).

ZIP4, which is encoded by the SLC39A4 gene, may be a novel biomarker for PC: high expression of SLC39A4 correlates with low survival rate in patients with PC (41). We have listed the studies that have identified exosomal indicators of PC (**Table 1**) (32, 33, 35, 37, 38, 40–47). Further research is required to narrow down specific markers in the exosomes that can be used for early detection of PC.

**Table 1:** Studies analyzing specific exosomal proteins or nucleic acids associated with PC.

Study	Country/region	Specific protein	Specific nucleic acid	Cohort	Group	Sensitivity	Specificity	AUC	p value
Melo 2015 (32)	Germany	GPC1^+^ crExos	NA	PDAC (n = 190), healthy controls (n = 100), PCPL (n = 5), and BPD (n = 26)	1. PDAC (n = 190) and healthy controls (n = 100)	100%	100%	1	< 0.0001
					2. PCPL (n = 5) and healthy controls	100%	100%	1	< 0.0001
					3. PCPL(n = 5) and BPD (n = 26)	100%	100%	1	< 0.0001
Lai 2017 (37)	USA	NA	miR-10b,	PDAC (n = 29) and healthy controls (n = 6)	PDAC (n = 29) and healthy controls (n = 6)	100%	100%	1	< 0.001
			miR-21,			86%	100%	1	< 0.001
			miR-30c,			100%	100%	1	< 0.001
			miR-181a,			97%	100%	1	< 0.001
			low miR-let7a			93%	100%	1	< 0.001
Madhavan 2015 (42)	Germany	Panel of anti-CD44v6, -Tspan8, -EpCAM, and -CD104	NA	PC (n =131), healthy controls (n = 30), and other controls [CP patients (n = 25), benign pancreatic tumors (n = 22), non-PC patients (n =12)]	NA	96%	86%	NA	NA
		NA	miR-1246, miR-4644, miR-3976, and miR-4306			81%	94%	NA	NA
		Panel of anti-CD44v6, -Tspan8, -EpCAM, and -CD104	miR-1246, miR-4644, miR-3976, and miR-4306			100%	80%	NA	NA
Kitagawa 2019 (43)	Japan	NA	WASF2,	PC (n = 27), controls (n = 13), and patients with non-pancreatic diseases such as benign gastrointestinal diseases (n = xx)	NA	NA	NA	0.943	< 0.05
			ARF6,			NA	NA	0.94	< 0.05
			SNORA74A			NA	NA	0.909	< 0.05
			SNORA25			NA	NA	0.903	< 0.05
Xu 2017 (38)	USA	NA	miR-196a	Localized PC (Stage I-IIA, n =15), and healthy subjects (n = 15)	NA	NA	NA	0.81	0.0105
			miR-1246			NA	NA	0.73	0.0217
Wu 2020 (44)	China	NA	ex-miRNA-21	PC (n = 30) and CP (n = 10)	NA	NA	NA	0.869	0.003
			ex-miRNA-210			NA	NA	0.823	0.038
Pu 2020 (45)	China	NA	exmiR-21	PC (n = 36) and healthy controls (n = 65)	NA	NA	NA	0.717	0.0003
			ExmiR-21 and exmiR-10b			NA	NA	0.791	< 0.0001
Lewis 2018 (35)	USA	Glypican-1 and CD63	NA	PDAC (n = 20) and healthy subjects (n = 11)	NA	99%	82%	0.99	< 0.0001
Chen 2017 (46)	China	NA	miR-23b-3p	PC (n = 16), healthy controls (n = 20), and CP (n = 18)	NA	NA	NA	NA	< 0.05
Jin 2018 (41)	China	Zinc transporter ZIP4	NA	MP (n = 24), BP (n = 32), B (n = 32), and healthy controls (n = 46)	MP and N	NA	NA	0.893	< 0.0001
					MP and BP	NA	NA	0.89	< 0.0001
					MP and B	NA	NA	0.811	0.0053
Goto 2018 (40)	Japan	NA	miR-191	PC (n = 32), IPMN (n = 29), and healthy controls (n = 22)	PC and control	71.9%	84.2%	0.788	0.001
					IPMN and control	64.3%	79.0%	0.741	0.006
			miR-21		PC and control	80.7%	81.0%	0.826	< 0.001
					IPMN and control	75.9%	81.0%	0.741	0.004
			miR-451a		PC and control	65.6%	85.7%	0.759	0.002
					IPMN and control	62.1%	85.7%	0.742	0.004
Lux 2019 (33)	Germany	c-Met	NA	PDAC (n = 55), CP (n = 26), and benign serous cyst adenoma of the pancreas (n = 10)	NA	70%	85%	0.799	< 0.001
Wang 2021 (47)	China	NA	miRNA-1226	PDAC (n = 5) and BPs (n = 17)	PDAC and BPs	NA	NA	0.74	0.025

AUC, area under the receiver operatig characteristic curve; B, patients with biliary disease; BP, benign pancreatic disease; BPs, benign lesion of pancreas; BPD, benign pancreatic disease; c-Met, proto-oncogene mesenchymal-epithelial transition factor; CP, chronic pancreatitis; GPC1+ crExos, Glypican-1+ circulating exosomes; IPMN, intraductal papillary mucinous neoplasm; miR-10b, microRNA-10b; MP, malignant PC; NA, not applicable; PC, pancreatic cancer; PCPL, pancreatic cancer precursor lesions; PDAC, pancreatic ductal adenocarcinoma.

## Role of cfDNA and ctDNA in PC

cfDNA refers to DNA fragments in different body fluids, and they are present even in healthy individuals. When an individual suffers tissue damage or inflammatory reaction, or when an individual develops cancer, the level of cfDNA in the plasma increases (48). In contrast, ctDNA refers to DNA fragments that originate from the cells of primary or metastatic tumors and that enter the peripheral blood circulation.

Several studies have documented the potential use of ctDNA and cfDNA as prognostic and predictive biomarkers for the early diagnosis of PC. PDAC tissue typically shows extensive and heterogeneous mutations, with mutations in the KRAS gene being the most frequent. High expression of mutated KRAS has been linked to poor prognosis of PC. These mutational analyses have been performed using digital PCR, next-generation sequencing, combining single-strand library preparation and target capture (SLHC-seq) as well as endoscopic ultrasound-guided fine-needle aspiration biopsy tissue and KRAS amplicon-based deep sequencing. Mutations in various genes potentially linked to PC have been explored in ctDNA and cfDNA from plasma, serum, or tissue (**Tables 2**, **3**) (49–59).

**Table 2:** Subgroup analysis of genes associated with PDAC.

Study	Country/region	Gene	Cohort	Sensitivity	Specificity	AUC	p value
Lin 2018 (49)	China	KRAS mutations	PDAC (n = 65)	NA	NA	NA	<0.001
Takai 2016 (50)	Japan	KRAS	PDAC (n = 259)	NA	NA	NA	NA
Kinugasa 2015 (51)	Japan	KRAS	PDAC (n = 75)	NA	NA	NA	0.002
Shinjo 2020 (52)	Japan	ADAMTS2	PDAC (n = 37)	NA	NA	NA	0.31
		HOXA1		NA	NA	NA	0.98
		PCDH10		NA	NA	NA	0.07
		SEMA5A		NA	NA	NA	0.09
		SPSB4,		NA	NA	NA	<0.0001
Brychta 2016 (53)	Germany	KRAS mutations	PDAC (n = 50)	NA	NA	NA	<0.0001

AUC, area under the receiver operatig characteristic curve; ctDNA, circulating tumor DNA; NA, not applicable; PDAC, pancreatic ductal adenocarcinoma.

**Table 3:** Subgroup analysis of genetic elements associated with PDAC.

Study	Country/Region	Genetic element(s)	Cohort	Group	Sensitivity	Specificity	AUC	p value
Guler 2020 (54)	USA	5-hydroxymethylcytosine (5hmC) changes in circulating cfDNA	PDAC (n = 64) and HC (n = 243)	NA	NA	NA	0.92	NA
Bernard 2019 (55)	USA	KRAS MAF	PDAC (n = 34) and HC (n = 37)	NA	NA	NA	NA	0.0003
Adamo 2017 (56)	United Kingdom	KRAS, TP53, SMAD4, CDKN2A	PDAC (n = 26), CP (n = 14), and HC (n = 12)	PDAC and HC	NA	NA	NA	<0.05
				CP and HC	NA	NA	NA	<0.001
Berger 2016 (57)	Germany	Circulating GNAS and KRAS Mutations	IPM (n = 21), HC (n = 38), metastatic PDAC (n = 24), resected SCA (n = 26), borderline IPMN (n = 16)	IPMN and HC	NA	NA	NA	<0.001
				Metastatic PDAC and HC	NA	NA	NA	<0.0001
				Resected SCA and HC	NA	NA	NA	0.0005
				Borderline IPMN and HC	NA	NA	NA	0.001
Liu 2019 (58)	China	KRAS	PDAC (n = 113), HC (n = 28)	PDAC and HC	92%	100%	NA	< 0.01
				IPMN and HC	88%	100%	NA	<0.0001
				CPC and HC	83%	100%	NA	<0.001
Wang 2019 (59)	China	KRAS MAF	PDAC (n = 110) and PB (n = 52)	PDAC and PB	42%	100%	NA	<0.001

CP, chronic pancreatitis; GNAS,The GNAS gene codes for an alpha subunit of the guanine nucleotide-binding protein (G protein); HC, healthy controls; IPMN, intraductal papillary mucinous neoplasm; MAF, minor allele frequency; NA, not applicable; PB, pancreatic benign disease; PDAC, pancreatic ductal adenocarcinoma; SCA, Pancreatic Serous Cystadenoma.

Using multiplex droplet digital PCR, Takai et al. (50) retrospectively analyzed KRAS mutations in the plasma cfDNA of 259 patients with PDAC. Those researchers worked with a modified Sure Select Kapa Illumina platform and an original panel of 60 genes to perform cfDNA deep sequencing on 48 patients, who showed a plasma KRAS mutation allele frequency ≥ 1%. Among 107 patients with inoperable tumors, 63 had KRAS mutations in plasma cfDNA. The corresponding mutations were also identified in the DNA of healthy tissues from 14 of 48 patients examined. In addition, the level of cfDNA in blood correlated with the presence of PC. These results suggest that detection of plasma cfDNA mutations can help diagnose PDAC.

Liu et al. (58) used single-strand library preparation and hybrid-capture-based cfDNA sequencing to analyze cfDNA fragments in PC patients. They found that analyzing short or damaged cfDNA fragments increased the sensitivity and accuracy of ctDNA detection. Wang et al. (59) showed that determining the minor KRAS allele frequency in ctDNA could reveal information for staging PC, and that assaying both mutant KRAS ctDNA and CA19-9 could improve the sensitivity of early diagnosis of PC. Shinjo et al. (52) developed a method to detect DNA methylation in cfDNA samples, which is based on the enrichment of methyl CpG binding (MBD) protein and digital PCR (MBD – DDPCR). With their method, they were able to detect at least one of five markers of DNA methylation in 23 of 47 PC patients. More than 80% of the regions methylated in the cfDNA overlapped with methylated regions identified in tumor tissue, and the two methylation patterns correlated strongly with each other (r = 0.97). Thus, the use of five methylation markers and KRAS mutations may help detect PC.

Lin et al. (49) also investigated KRAS mutations in ctDNA as a potential diagnostic tool for PDAC patients who had undergone irreversible electroporation. Among the 65 cases, ctDNA was detected in 20 (29.2%), and their median overall survival was significantly shorter than that of ctDNA-negative patients (11.4 vs. 14.3 months). Adamo et al. (56) performed targeted next-generation sequencing of 50 oncogene mutation hotspots using samples from 26 patients with PDAC, 14 patients with chronic pancreatitis, and 12 healthy controls. They found that the median total cfDNA levels were higher in PDAC patients than in controls or patients with chronic pancreatitis, and that KRAS mutations were significantly associated with low survival. This study suggests that cfDNA analysis and specifically KRAS mutational analysis can provide prognostic information in PC, although not necessarily diagnostic information.

Another study compared the KRAS mutations between tissue DNA and ctDNA of 75 patients with PC (51). KRAS mutation rates were 74.7% in tissues and 62.6% in ctDNA, and the two sets of results were consistent in 77% of patients. The survival rate of patients with KRAS mutations in ctDNA was significantly lower than that of patients without mutations, although survival did not differ significantly between patients who had KRAS mutations or not in their tissue DNA. The survival difference was particularly large in the case of G12V mutations. These results support ctDNA analysis for the diagnosis of PC and prediction of survival rate. Similarly, Brychta et al. (53) reported that 72% of patients with pancreatic tumors were positive for KRAS mutations, 44% were positive for G12D, 20% for G12V, and 10% for G12C; one tumor was positive for both G12D and G12V. A detailed analysis of the mutations in matched plasma samples showed a detection rate of 44% for G12D, 50% for G12V, and 0% for G12C. The 20 healthy controls did not show any KRAS mutations.

5-hydroxymethylcytosine (5hmc) is an epigenetic marker in cfDNA that has been used as a non-invasive marker for the early detection of PDAC (54). Changes in 5hmc levels may help classify PDAC, even in early stages of the disease, since changes in these levels have been linked to alterations in PDAC-associated genes (54).

Guler et al. (54) showed significantly higher cfDNA levels in the PDAC cohort than in the healthy cohort. Among thousands of genes associated with PDAC, the most significant ones are related to pancreatic development or function (GATA4, GATA6, Prox1, Onecut1, Meis2) and cancer pathogenesis (Yap1, Tead1, Prox1, IGF1) (51, 53, 57–59). Another study conducted by Berger et al. (60) detected GNAS, the gene codes for an alpha subunit of the guanine nucleotide-binding protein (G protein) mutations in cfDNA from patients with intraductal papillary mucinous neoplasms, but not in patients with serous cystadenoma or healthy controls. They found that the total amount of cfDNA may be useful for diagnosis of PDAC and predicting prognosis.

To investigate the clinical value of ctDNA and exoDNA in PC, Bernard et al. (55) collected fluid biopsy samples from patients with localized or metastatic PC, and found that in patients with potentially resectable tumors, an increase in the levels of exosome Deoxyribo Nucleic Acid (exoDNA) after neoadjuvant therapy correlated with disease progression, whereas levels of ctDNA did not correlate with prognosis. The concordance of KRAS mutations between surgically resected tissues and liquid biopsies was > 95%.

Studies have also explored the prognostic role of other biomarkers, such as miRNA (61) and the gene encoding thymidylate synthase (62). However, the sensitivity and specificity of these biomarkers for detecting PC are unclear.

As methods to analyze ctDNA and cfDNA advance, it may become possible to rely on liquid biopsy to identify cancer early, even in asymptomatic individuals, allowing timely interventions that can improve survival. After radical surgery, cfDNA in postoperative plasma collected within a few weeks after surgery can be analyzed to determine whether mutations or other changes known to exist in the resected tumors persist. Analysis of ctDNA and cfDNA can reveal genetic and epigenetic changes that can aid diagnosis and prediction of prognosis. Further clinical trials with large samples are required to continue advancing liquid biopsy technology and thereby pave the way for the development of precision oncology approaches to treat this deadly disease.

## Role of CTCs in PC

CTCs are cancer cells that derive from primary or metastatic tumors and that can be isolated directly from tumors or detected in the peripheral blood (63–65). CTC detection usually involves enriching for specific CTCs based on geno- or phenotyping (66). CTCs are observed in the peripheral blood of patients with all types of cancer, but rarely in healthy individuals or those with non-malignant diseases (67). CTCs are identified based on fluorescence *in situ* hybridization with a chromosome 8 centromere (CEP8) probe. Based on the copy number of CEP8, the CTCs are classified as triploid, tetraploid, pentaploid, or polyploid (68). The above CTCs can also be detected in peripheral blood of healthy people, but the number is far less than that of PC. It may be related to stressors such as oxidative damage, hypertension and aging (69, 70). CTCs have been used for the early screening and diagnosis of PC (**Table 4**) (68, 71, 74). For example, Ankeny et al. (61) studied 72 patients with PDAC and found that the CTCs in peripheral blood were round/ovoid, ≥ 6 μm in size, and positive for DAPI, CK and CEA, but negative for CD45. Based on CTCs, those investigators were able to distinguish between patients with local/regional tumors (I-III, n = 45) and those with metastatic tumors (IV, n = 27).

**Table 4:** Performance of different phenotypes of CTCs for diagnosing pancreatic cancer.

Study	Country/Region	Gene locus	Cohort	Cut-off value	Sensitivity	Specificity	AUC	p value
Liu 2017 (68)	China	Cells with features of CD45-/DAPI+/CEP8 > 2 were detected as CTCs	PDAC (n = 95) and HC (n = 48)	2 CTC/3.2 mL	75.8%	68.7%	0.791	<0.001
Ankeny 2016(71)	USA	CTCs were defined as round/ovoid, size ≥ 6 μm, DAPI+/CD45-, and CK+ or CEA+	PDAC (n = 72) and non-adenocarcinoma diagnoses (n = 28)	1 CTC/4 mL	75%	96.4%	0.867	<0.001
			Local/regional tumors (n = 45), and metastatic tumors (n = 27)	3 CTC/4 mL	85.2%	86.7%	0.885	<0.001
Xu 2017 (38)	China	Type-A phenotype: CK18−, CD45−, DAPI+, CEP-8 = 3 Type-B phenotype: CK18+, CD45−, DAPI+ Type-E phenotype: CTMs	PC (n = 40) and controls, including benign tumor of the pancreas (n = 8) and HC (n = 35)	1.5 CTC/7.5 mL	77.5%	79.1%	0.861	<0.0001
Wei 2019 (72)	China	Vimentin CTCs and CA19-9	PDAC (n = 100) and HC (n = 30)	NA	NA	NA	0.968	NA
Cheng 2020 (73)	China	FR^+^ CTCs and CA19-9	PC (n = 45), and patients with benign pancreatic diseases (n = 6)	NA	97.8%	83.3%	0.944	<0.001

AUC, area under curve; CD45, cluster of Differentiation 45; CEA, carcinoembryonic antigen; CEP8, centromere probe 8; CK, cytokeratin; CTCs, circulating tumor cells; CTM, circulating tumor microemboli; DAPI, 4',6-diamidino-2-phenylindole; FR+ CTCs, folate receptor positive circulating tumor cells. HC, healthy control; NA, not applicable; PC, pancreatic cancer; PDAC, pancreatic ductal adenocarcinoma.

Other studies have reported that a combination of CTC analysis and CA19-9 assay can improve the diagnostic power of CA19-9 (**Table 4**) (72, 73). One problem with CA19-9 is that it is absent from about 10% of the general population, who therefore do not express CA 19-9 even if they develop PC. In addition, CA19-9 is not specific for PC: it can also be detected in association with obstructive jaundice (acute cholangitis and cholangiolithiasis) and malignant diseases (colorectal cancer, gastric cancer, bladder cancer and uterine squamous cell carcinoma). CTC levels may confer some specificity, since they are unaffected by bilirubin levels, which do affect CA 19-9 levels (64). Indeed, combining analysis of CTCs and CA19-9 allowed diagnosis of PC with a sensitivity of 97.5% in one study (74). Further studies should explore the potential of CTCs for diagnosing PC.

## Role of Circulating miRNAs in PC

MicroRNAs are non-coding, single-stranded RNA molecules as long as 22 nucleotides that act as posttranscriptional regulators of gene expression and thereby control many key cellular processes (75, 76). Circulating miRNAs have been used as markers in liquid biopsy. For example, miR-223 (77), miR−23b−3p (46), a six-miRNA signature (78), miR-100 (79), miR-205 (80), miR-192-5p (81), a six-miRNA panel (82), and miR-483-3p (83) have been used to discriminate PDAC patients from healthy controls or patients with chronic pancreatitis (**Table 5**). Trager et al. (68) showed that, compared to CA 19-9 alone (AUC 0.854, 95% CI 0.763-0.944), a combination of serum miR-205 and CA19-9 had significantly better diagnostic potential for PDAC (AUC 0.917, 95% CI 0.818-1.02). Similarly, Shao et al. (71) showed that, compared to CA 19-9 alone (AUC 0.87, 95% CI 0.79-0.94), a combination of serum miR-483-3p and CA19-9 showed superior diagnostic performance for PDAC (AUC 0.94, 95% CI 0.89-0.99).

**Table 5:** Performance of circulating microRNAs for diagnosing pancreatic cancer.

Study	Country/Region	Cohort	Genetic element(s)	Group	Sensitivity	Specificity	AUC	p value
Komatsu 2015 (77)	Japan	PC (n = 71) and HC(n = 67)	MiR-223	NA	NA	NA	NA	<0.0001
Chen 2017 (46)	China	PC (n = 16), HC (n = 20), and CP (n = 18)	MiR-23b-3p	PC (n = 16), healthy controls (n = 20), or CP (n = 18)	NA	NA	NA	<0.05
Zhou 2018 (78)	China	PC (n = 112) and HC (n = 116)	Six-miRNA signature:miR-122-5p, miR-125b-5p, miR-192-5p, miR-193b-3p, miR-221-3p and miR-27b-3p	NA	79.8%	75.7%	0.833	NA
Stroese 2018 (79)	Germany	PDAC UICC Stages I-IV (n = 90), CP (n = 40), and HC (n = 40)	miR-100	PDAC and HC	NA	NA	0.81	<0.001
				PDAC and CP	NA	NA	0.64	0.061
				HC and CP	NA	NA	0.73	0.014
			miR-99b	PDAC and HC	NA	NA	0.76	<0.001
				PDAC and CP	NA	NA	0.55	0.482
				HC and CP	NA	NA	0.72	0.009
			miR-99a	PDAC and HC	NA	NA	0.72	0.002
				PDAC and CP	NA	NA	0.68	0.011
			miR-21	PDAC and HC	NA	NA	0.71	0.005
				PDAC and CP	NA	NA	0.70	0.005
Michael Traeger 2018 (80)	Germany	PDAC UICC Stages II-IV (n = 65), CP (n = 32), and HC (n = 34)	miR-205	PDAC and HC	NA	NA	0.722	0.008
				PDAC and CP	NA	NA	0.671	0.051
				HC and CP	NA	NA	0.612	0.282
			miR-205 and CA19-9	PDAC and HC	NA	NA	0.917	0.052
				PDAC and CP	NA	NA	0.885	0.001
				HC and CP	NA	NA	0.944	0.059
Flammang 2020 (81)	Germany	PDAC UICC Stages II-IV (n = 44), CP(n = 11), and HC(n = 12)	Exosomal miR-192-5p	PDAC and HC	NA	NA	0.83	0.0004
				PDAC and CP	NA	NA	0.54	0.7206
				HC and CP	NA	NA	0.80	0.0164
		PDAC UICC Stages II-IV (n = 42), CP (n = 13), and HC (n = 14)	Circulating miR-192-5p	PDAC and HC	NA	NA	0.64	0.1208
				PDAC and CP	NA	NA	0.64	0.1272
				HC and CP	NA	NA	0.55	0.6275
Zou 2019 (82)	China	PC (n = 159) and HC (n = 137)	Six‐miRNA panel: let-7b-5p; miR-192-5p; miR-19a-3p; miR-19b-3p; miR-223-3p; and miR-25-3p.	Combination of two cohorts - training and testing phases (129 PC and 107 HCs)	95.3%	76.7%	0.91	NA
				External validation phase (30 PC and 30 HCs)	93.3%	96%	0.978	NA
Shao 2021 (83)	China	PDAC AJCC Stages I-IV (n = 63), CP (n = 40), and HC (n = 22)	Serum miR-483-3p	PDAC and HC	74.6%	77.3%	0.81	<0.0001
				PDAC (tumor size ≤ 2 cm, n = 20) and HC	85.7%	72.7%	0.83	NA
				PDAC(stage I, n = 18) and HC	72.2%	72.7%	0.79	NA
			Exosomal miR-483-3p	PDAC and HC	NA	NA	0.69	<0.01
			Serum and exosomal miR-483-3p	PDAC and HC	NA	NA	0.84	NA

AUC, area under curve; AJCC, the American Joint Committee on Cancer; CA19-9, carbohydrate antigen 19-9; CP, chronic pancreatitis; HC, healthy control; NA, not applicable; PC, pancreatic cancer; PDAC, the pancreatic ductal adenocarcinoma; UICC, Union international control cancer.

Several studies have reported miR-100, miR-192-5p, miR-483-3p and a six-miRNA signature containing miR-125b-5p as potential prognostic markers in PDAC patients. Zhao et al. (66) showed that the downregulation of plasma miR-125b-5p may predict worse overall survival, independently of tumor stage and CA19-9 expression. Stroese et al. (78) showed that low expression of circulating miR-100 is associated with significantly better overall survival and recurrence-free survival (79). At the same time, Flammag et al. (69) showed that overexpression of miR-192-5p in patients after therapeutic resection is associated with longer overall survival and delayed recurrence (81), while high levels of exosomal miR-483-3p predict poor prognosis (83). Therefore, miR-125b-5p and miR-192-5p may be protective factors against PC, while miR-100 and miR-483-3p may be risk factors. The diverse roles played by circulating miRNAs in PC must be explored further in order to clarify their diagnostic and prognostic value.

## Combination of Biomarkers

A number of studies have explored combinations of biomarkers as potential diagnostic tools for PC (**Table 6**) (84–88). Cohen et al. (84) found that a combination of CA19-9, CEA, HGF, OPN, and KRAS mutations in ctDNA was more powerful for the early diagnosis of PDAC than each of the five markers on their own. Eissa et al. (85) detected BNC1 and ADAMTS1 in 100% of PC patients with stage I cancer, 88.9% of patients with stage IIA cancer, and 100% of patients with stage IIB cancer. Using both genes, those researchers were able to identify patients with stage I or II cancer with sensitivity of 94.8% and specificity of 91.6%, suggesting their usefulness for detecting early-stage PDAC.

**Table 6:** Performance of biomarker combinations for diagnosing pancreatic cancer.

Study	Country/Region	Test index	Cohort	Group	Cut-off value	Sensitivity	Specificity	AUC	p value
Cohen 2017 (84)	USA	ctDNA KRAS mutation, CEA, CA19-9, HGF, and OPN	PDAC AJCC stages I-II (n = 221), HC (n = 182)	NA	CA19-9 (100 U/mL), CEA (7.5 ng/mL), HGF (0.92 ng/mL), or OPN (158 ng/mL)	64%	99.5%	NA	NA
Eissa 2019 (85)	USA	Genes: BNC1 and ADAMTS1	PDAC (Stage I = 8, Stage OOa = 9, Stage IIb = 20, Stage III/IV = 2), and HC (n = 95)	NA	NA	97.4%	91.6%	0.95	NA
Berger 2019 (86)	Germany	CA19-9, THBS2, and cfDNA	PDAC (n = 52) and control group, including IPMN (n =15) and pancreatitis (n = 32)	PDAC and control group	CA19-9 (≥ 55 U/ml), THBS2 (≥ 42 ng/ml), cfDNA (16.2 ng/ml)	87%	92%	NA	NA
				PDAC (all stages) and control group	CA19-9 (≥ 55 U/ml), THBS2 (≥ 42 ng/ml), cfDNA (16.2 ng/ml)	NA	NA	0.94	0.0013
				PDAC stage I (n = 14) and control group	CA19-9 (≥ 55 U/ml), THBS2 (≥ 42 ng/ml), cfDNA (16.2 ng/ml)	NA	NA	0.9	0.0143
				PDAC stage II (n = 17) and control group	CA19-9 (≥ 55 U/ml), THBS2 (≥ 42 ng/ml), cfDNA (16.2 ng/ml)	NA	NA	0.96	0.1424
				PDAC stage III (n = 21) and control group	CA19-9 (≥ 55 U/ml), THBS2 (≥ 42 ng/ml), cfDNA (16.2 ng/ml)	NA	NA	0.94	0.0549
Yang 2020 (87)	Philadelphia	EV-CK18 mRNA, EV-CD63 mRNA, EV-miR.409, cfDNA, and CA19–9	PDAC (n = 57), HC (n = 49), and non-PDAC pancreatic disease (n = 30)	PDAC and non-cancer control	NA	88%	95%	0.95	0.103
Xiao 2020 (88)	China	Exosomal GPC1, CD82, and serum CA19-9	PDAC (n = 24), CP (n = 6), and HC (n = 26)	PDAC and HC	NA	65.38%	95.83%	0.942	0.2282
				PDAC and CP	NA	66.67%	95.83%	0.958	0.5467

AUC, area under curve; ADAMTS1, a disintegrin and metalloproteinase with thrombospondin motifs; AJCC, the American Joint Committee on Cancer; BNC1, zinc finger protein basonuclin-1; CD82, cluster of differentiation 82; CEA, carcinoembryonic antigen; CP, chronic pancreatitis; GPC1, glypican-1; HC, healthy control; HGF, hepatocyte growth factor; IPMN, intraductal papillary mucinous neoplasia; NA, not applicable; OPN, osteopontin; PC, pancreatic cancer; PDAC, pancreatic ductal adenocarcinoma; THBS2, thrombospondin-2.

Berger et al. (86) found that a combination of CA19-9, THBS2, and cfDNA can be used to distinguish PDAC patients from healthy controls, and that the combination identified patients with stage I-III cancer significantly better than each biomarker on its own. Yang et al. (87) found that combining extracellular tumor miRNAs and mRNAs, cfDNA, CA19-9, and imaging techniques allowed differentiation of PDAC patients from healthy controls as well as differentiation of local (M0) from metastatic (M1) cancer. The combination of methods was superior to imaging alone. Xiao et al. (88) found that a combination of exosomal GPC1, CD82, and serum CA19-9 can distinguish patients with PDAC from healthy controls or patients with chronic pancreatitis.

Although the studies presented here emphasize the power of combining biomarkers to compensate for the relatively low diagnostic value of each marker individually, using biomarker combinations can complicate early detection of PC, especially because it may be very difficult to measure all biomarkers with a single instrument or method. Therefore, further research must be conducted to find a way to integrate the diagnostic potential of different biomarkers.

## Precursor Lesions of PC

Precursor lesions of PDAC including pancreatic intraepithelial neoplasias, intraductal papillary mucinous neoplasms, intraductal tubulopapillary neoplasms, intraductal oncocytic papillary neoplasms, and mucinous cystic neoplasms (89, 90). In addition to intraepithelial neoplasias, the above precursor lesions can be detected by CT, MRI or endoscopic ultrasound. However, imaging may not be able to accurately distinguish the type of lesion or important histological features, which is difficult to predict the next progression (91).

Chronic pancreatitis is considered to be closely related to early lesions of PC: chronic pancreatitis is associated with higher CA19-9 levels and higher risk of PDAC (92). However, the relationship between chronic pancreatitis and PC is poorly understood (93). Lai et al. (37) demonstrated that PDAC patients had higher levels of miR-10b, miR-21, miR-30c, and miR-181a, but lower levels of miR-let7a, than healthy controls or patients with chronic pancreatitis. PC patients show higher levels of circulating miR-21 and miR-210 than those with chronic pancreatitis (44), suggesting that exosomal miR-21 may distinguish between patients with early- or late-stage PC as well as between patients with PC and healthy individuals. Using a combination of biomarkers that include exosomal miR-21 and miR-10b may further increase diagnostic performance (45).

If clinicians use liquid biopsy to detect early signs of malignant tumor, precursor lesions, and chronic pancreatitis, patients can be treated and monitored as soon as possible in order to improve their quality of life and prolong survival.

## Perspective

Early detection of PC can enhance the probability that a patient is eligible for surgery, which can improve prognosis (94). The NCCN guidelines state that PC screening should be conducted on individuals with a family history of PC, regardless of whether the individuals have clinical symptoms (2). For most populations, imaging-based screening techniques are not feasible since they are expensive and invasive. In particular, it is not cost-effective to screen the many people with PC risk factors, which include a history of smoking or diabetes mellitus. Therefore, we believe that screening using liquid biopsy can be extremely beneficial for expanding the scope of screening efforts. The present review summarizes advances in liquid biopsy-based assays of circulating exosomes, ctDNAs, CTCs, and miRNAs for early detection of PC.

The various liquid biopsy markers in the literature have advantages and disadvantages. The methods for CTC identification and enrichment from peripheral blood need to be improved. When analyzing ctDNA, it can be difficult to discriminate between material from tumors or non-tumor tissue, and tumor exosomes are challenging to purify. The clinical application of each potential biomarker and its corresponding characteristics have been analyzed more broadly in another review (26), and the present work focuses on advances in liquid biopsy research for early diagnosis of PC. We conclude that liquid biopsy can be useful for this purpose, and future research should identify the optimal biomarker combinations among circulating exosomes, ctDNA, CTCs, and miRNA. Metabolomic biomarkers also exist in blood, urine and even saliva. The combination of multiple metabolites showed high diagnostic value (95), such as amino acids (96, 97), taurine (98), creatine and glutamine (99). Kobayashi et al. (100) conducted a study on 43 PC patients and 42 healthy volunteers using a gas chromatography-mass spectrometry metabonomics model. The results showed that metabolites (AUC = 0.928) were more accurate than conventional CA19-9 (AUC = 0.824) and CEA (AUC = 0.799). In addition, the model has high sensitivity (86.0%) and specificity (88.1%) for PC, which is not inferior to traditional markers. Another limitation that limits the widespread use of liquid biopsy is that there are a very few studies comparing different biomarker side by side. Future studies that overcome this limitation would have more clinical achievements.

The only effective way to improve the prognosis of PC patients is to conduct pancreatectomy at an early stage. Therefore, it is crucial to diagnose PC or its precursor lesions as early as possible. The biggest advantages of liquid biopsy in the early diagnosis of cancer are its non-invasiveness, reproducibility, and suitability for low-cost screening. Although several studies have demonstrated the sensitivity and specificity of certain biomarkers in liquid biopsy, most of the studies have a small sample size (e.g. n < 100), finding from which may be accidental and not representative. Therefore, further work is needed to validate the biomarkers in the clinic, preferably involving large, multi-center samples. Additionally, the costs and technical feasibility of simultaneously assaying multiple biomarkers in liquid biopsies should be optimized.

While it is laudable that NCCN guidelines acknowledge the application of liquid biopsy in PC, the guidelines should be updated to reflect the latest advances in the literature. In particular, strong clinical evidence already exists that GPC1 stand a good chance of meeting the requirements of an effective screening marker of PC. Such information should be mentioned in the NCCN guidelines so that more researchers can notice this biomarker. Researchers should also undertake clinical studies and meta-analyses to provide high-level evidence for this and other potential PC biomarkers in liquid biopsy.

## Conclusion

In the future, it may be possible to rely on liquid biopsy to help in the differential diagnosis of chronic pancreatitis and PC. Of course, liquid biopsy may also become useful for the early diagnosis of liver, gastric, and breast cancers. The far-reaching potential of liquid biopsy for early cancer diagnosis and for prediction of prognosis argue for new lines of investigation to clarify the roles of potential biomarkers in disease onset and progression. The resulting insights will give patients more access to life-extending treatments and help clinicians personalize treatment plans. Growing evidence suggests that liquid biopsy can be an effective technique for the early diagnosis of PC, which would allow earlier initiation of treatment that can prolong survival.

More importantly, we must (i) standardize our detection methodologies, (ii) compare different biomarker in the same cohorts, (iii) combine our sample cohorts in order to analyze reasonable numbers (everything below n = 100 is maybe “new” but remains anecdotal).

## Author Contributions

J-HZ conceptualized the study. All authors drafted and revised the manuscript. All authors contributed to the article and approved the submitted version.

## Funding

This work is partly supported by the National Natural Science Foundation of China (82060510), the ‘Guangxi BaGui Scholars’ Special Fund (2019AQ20), the Guangxi Natural Science Foundation (2018GXNSFBA138018 and 2020GXNSFAA159022), the 2021 Undergraduate Innovation and Entrepreneurship Training Program of Guangxi Medical University (202110598038), and Guangxi Key Research and Development Program (GuikeAB18126055).

## Conflict of Interest

The authors declare that the research was conducted in the absence of any commercial or financial relationships that could be construed as a potential conflict of interest.

## Publisher’s Note

All claims expressed in this article are solely those of the authors and do not necessarily represent those of their affiliated organizations, or those of the publisher, the editors and the reviewers. Any product that may be evaluated in this article, or claim that may be made by its manufacturer, is not guaranteed or endorsed by the publisher.
